# The Rheumatoid Hand

**Published:** 2013-02-11

**Authors:** Edward Hahn, Earl Fleegler

**Affiliations:** Division of Plastic and Reconstructive Surgery, New Jersey Medical School, University of Medicine and Dentistry of New Jersey, Newark

**Figure:**
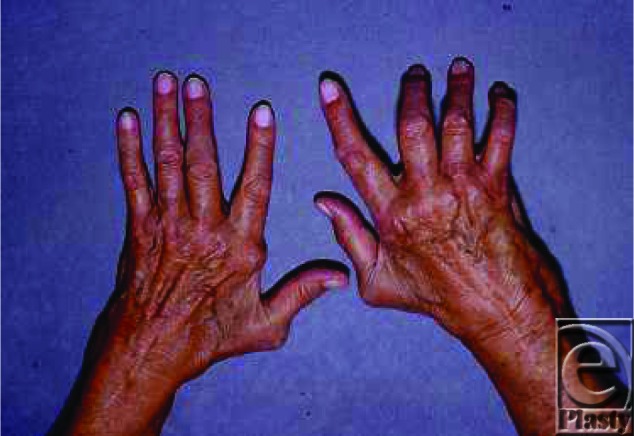


## DESCRIPTION

A 70-year-old woman presents with chronic bilateral wrist and hand pain, difficulty with activities of daily living, and progressive deformities in her hands.

## QUESTIONS

1. **What is the pathophysiology of rheumatoid arthritis?**
2. **Describe the evaluation of the patient with suspected rheumatoid arthritis.**
3. **What are the radiologic findings of rheumatoid arthritis?**
4. **Why do tendons rupture in the rheumatoid hand?**
5. **What is the characteristic deformity of the wrist, metacarpal phalangeal joints, proximal interphalangeal joints, and distal interphalangeal joints**

## DISCUSSION

Rheumatoid arthritis (RA) is a chronic systemic inflammatory disease that affects approximately 1.3 million Americans. Seventy-five percent of these patients are women. The age of onset is most often between the fourth and sixth decades of life and is insidious in nature.

Rheumatoid arthritis is characterized by progressive joint destruction and deformity that can lead to significant loss of hand function and impaired ability to perform activities of daily living. The pathogenesis of RA is not completely understood. Synovial hyperplasia, as well as synovitis and tenosynovitis caused by inflammatory cell infiltration are pathologic processes observed in RA. The synovium expands and develops into a pannus; this causes destruction of joint capsules and ligaments. Lytic destruction of the articular cartilage causes periarticular osteopenia. Cartilage, ligaments, and tendons are vulnerable to the inflammatory process. Synovitis of the wrist begins at radiocarpal and distal radioulnar joint (DRUJ). Destruction of the DRUJ leads to caput ulnae syndrome: dorsal dislocation of the distal ulna, supination of the carpus on the radius, and volar subluxation of the extensor carpi ulnaris tendon. The “piano keyboard sign” is indicative of DRUJ instability and is elicited when the ulnar head is depressed volarly and rebounds as pressure is released. A “Z collapse pattern” is observed as the carpus translates ulnarly, the metacarpals deviate radially, followed by ulnar deviation of the fingers. Distortions of the fingers are often in the form of swan-neck and boutonniere deformities. Patients with these finger deformities often possess unilateral intrinsic muscle tightness on the ulnar aspect of the finger causing an ulnar drift of the digits. Laxity of the volar plate and the collateral ligaments allow for dislocation of the flexor tendon volarly and ulnarly—contributing to the ulnar drift. The presence of ulnar drift improves proximal interphalangeal (PIP) joint flexion. The thumb often deforms with contraction of the metacarpophalangeal (MCP) joint and extension of the PIP joint.

The mechanisms of tendon rupture include attrition of the tendon over rough bony prominences, ischemia due to the expanding synovium, and infiltration (tenosynovitis). Rupture of the ulnar extensor tendons usually precedes rupture of the radial extensor tendons and is often due to caput ulnae syndrome. This phenomenon is known as Vaughn-Jackson Syndrome. The extensor pollicis longus may rupture on Lister's tubercle. Extensor tendon rupture is more common than flexor tendon rupture. The flexor pollicis longus tendon is the most common flexor tendon ruptured and is known as a Mannerfelt lesion. Rupture of the flexor pollicis longus tendon is often due to a volar bony scaphoid spur.

The work-up for rheumatoid arthritis includes laboratory, radiographic, clinical history, and physical examination findings. While there are no pathognomonic tests for RA, anti-CCp antibodies and rheumatoid factor are highly specific for RA. Erythrocyte sedimentation rate, C-reactive protein, antinuclear antibody assay can assist in deducing the diagnosis. Radiographic views of the hands, wrists, feet, and cervical spine may demonstrate marginal joint erosion, periarticular osteopenia, joint subluxation, and joint ankylosis. Symmetric polyarthritis that affects hands and feet are the trademark features of RA. Unlike osteoarthritis, MCP and PIP joints are more commonly affected than distal interphalyngeal joints. Enlargement of the PIP joint known as “Bouchard's nodes” may be observed. Rheumatoid nodules noted over the bony prominences of the extensor surfaces may be present. Before experiencing joint pain and swelling, patients may experience symptoms of fatigue, fever, arthralgia, and weakness. Patients often complain of morning joint stiffness and pain that lasts more than 1 hour. Other joints such as the cervical spine, and extra-articular involvement of organs such as the skin, heart, lungs, and eyes can exist.

The primary goals in treating the rheumatoid hand are pain relief, restoration of function, and cosmetic improvement of the hand. Deformation, in and of itself, is not an indication for surgery. Many patients experience joint deformation without pain or dysfunction. Patients who have failed medical management and exhibit impaired ability to perform activities of daily living may be surgical candidates. RA may involve the cervical spine in 60% to 90% of patients. Radiographic and clinical investigation of cervical instability is necessary prior to any surgical intervention. Surgical treatment of the rheumatoid hand is complex and requires an experienced surgeon. Should surgery be indicated, it is advisable to address the lower extremities prior to the upper extremities. Furthermore, proximal joints should be treated before distal joints, elbow before wrist, and wrist before MCP joint. Surgical treatment options for the rheumatoid wrist includes synovectomy of radiocarpal joint and DRUJ, partial arthrodesis, ulnar resection, DRUJ fusion, wrist arthroplasty, and total wrist arthrodesis. Surgical treatment options of rheumatoid swan neck deformity include flexor PIP tenodesis, intrinsic release, MCP joint realignment, arthrodesis, and arthroplasty. Boutonniere deformities can be treated with synovectomy, central slip reconstruction, lateral band reconstruction, PIP fusion, or arthroplasty.
